# Correction: The combination of mupirocin and *Kayvirus* broadens the decolonization effect against *Staphylococcus aureus*

**DOI:** 10.1007/s00253-026-13900-3

**Published:** 2026-06-04

**Authors:** Alena Siváková, Eliška Figallová, Tibor Botka, Lukáš Vacek, Jan Tkadlec, Jan Vrbský, Martin Osowski, Petr Petráš, Dominika Polaštík Kleknerová, Milada Dvořáčková, Pavlína Urbanová, Roman Pantůček, Filip Růžička

**Affiliations:** 1 Department of Microbiology, Faculty of Medicine, Masaryk University and St. Anne’s University Hospital, Brno, Czech Republic; 2 Department of Experimental Biology, Faculty of Science, Masaryk University, Brno, Czech Republic; 3 Department of Medical Microbiology, Second Faculty of Medicine, Charles University and University Hospital Motol and Homolka, Prague, Czech Republic; 4 National Reference Laboratory for Staphylococci, National Institute of Public Health, Prague, Czech Republic

**Correction: Applied Microbiology and Biotechnology (2026) 110:157**

10.1007/s00253-026-13814-0

In this article, the author’s name Eliška Figallová was incorrectly written as Eliška FigallováPolaštík Kleknerová in the web version of the publication.

The original article has been corrected.

